# REPRIEVE final results: What does it mean for guidelines in low‐ and middle‐income countries?

**DOI:** 10.1002/jia2.26525

**Published:** 2025-06-10

**Authors:** Simiso Sokhela, Jennifer M. Manne‐Goehler, Samanta Lalla‐Edward, Mark J. Siedner, Mohammed K. Ali, Andrew Hill, Aaloke Mody, Anton Pozniak, Jeremy Nel, W. D. Francois Venter

**Affiliations:** ^1^ Wits Ezintsha Faculty of Health Sciences University of the Witwatersrand Johannesburg South Africa; ^2^ Division of Infectious Diseases Brigham and Women's Hospital Boston Massachusetts USA; ^3^ Department of Medicine Harvard Medical School Boston Massachusetts USA; ^4^ Clinical Research Department Africa Health Research Institute KwaZulu‐Natal South Africa; ^5^ Faculty of Medicine University of KwaZulu‐Natal Durban South Africa; ^6^ Hubert Department of Global Health Rollins School of Public Health Emory University Atlanta Georgia USA; ^7^ Department of Family and Preventive Medicine School of Medicine Emory University Atlanta Georgia USA; ^8^ Department of Translational Medicine Liverpool University Liverpool UK; ^9^ Division of Infectious Diseases, Department of Medicine St. Louis School of Medicine Washington University St Louis Missouri USA; ^10^ Chelsea and Westminster Hospital and London School of Hygiene and Tropical Medicine London UK; ^11^ Division of Infectious Diseases Faculty of Health Sciences University of the Witwatersrand Johannesburg South Africa; ^12^ Department of Public Health Medicine School of Health Systems and Public Health Faculty of Health Sciences University of Pretoria Pretoria South Africa

**Keywords:** cardiovascular, HIV, MACE, pitavastatin, REPRIEVE, statin

## Abstract

**Introduction:**

The REPRIEVE study demonstrated significant reductions in major adverse cardiovascular events (MACE) with pitavastatin among people living with HIV (PWH) with low to moderate cardiovascular risk. Most MACE events occurred in higher‐income countries, raising important considerations for similar primary prevention interventions within HIV programmes in low‐ and middle‐income countries (LMICs) as antiretrovirals become safer and as PWH age.

**Discussion:**

Limited data from Africa and within REPRIEVE suggests that MACE may not be as prevalent among PWH as within other geographies. Consequently, there remain questions about the appropriateness of extrapolating REPRIEVE data to the region and whether it should motivate programmatic implementation on the continent. Moreover, glucose and lipid screening used in REPRIEVE raise concerns about additional resources for similar screening, where there is little existing infrastructure and subsequent treatment. Similarly, questions around funding priorities, and health worker resource allocation for MACE prevention, particularly in the context of competing health priorities and limited health financing, need to be addressed. Newer cardiovascular medications, with cardiac, renal, hepatic, diabetes and weight loss benefits, may have greater promise, although cost remains a major concern. Finally, successful implementation with statins or other proven interventions will be unlikely, unless systemic change within non‐communicable disease health system delivery programmes occurs first. However, HIV programmes and public health systems more generally have shown themselves to be poor at screening and treating other cardiovascular risk factors, including aspects as simple as raised blood pressure, even in high‐income countries, and statins remain grossly under‐prescribed for primary and secondary prevention internationally.

**Conclusions:**

REPRIEVE turned a spotlight on how ill‐prepared current HIV programmes are to implement the simplest and safest primary care prevention interventions for cardiometabolic disease within LMICs. As data for existing and new interventions become available, HIV delivery systems will need to raise their standard beyond simply prescribing antiretrovirals and taking viral loads.

## 1 INTRODUCTION

REPRIEVE, a major and keenly anticipated study in the HIV community, reported final results in April 2024, with 32 endpoints added to the initial 225 in the preliminary report, but no significant change to the conclusion [1, 2]. The study demonstrated that taking pitavastatin, a moderate‐intensity statin with no major drug interactions with modern antiretrovirals, decreased major adverse cardiovascular events (MACE) in low‐ to moderate‐risk participants with HIV.

REPRIEVE is an achievement on many fronts, other than being the largest randomized trial among participants living with HIV ever conducted. Investigator‐driven studies are hard enough to perform, but doing them at this scale, across 12 countries, during an unprecedented parallel COVID‐19 pandemic, while both HIV and cardiovascular disease prevention guidelines change over the course of the study, was remarkable. The REPRIEVE team had broad recruitment criteria and included a greater proportion of women and Black participants, those in low‐income settings, and those with hepatitis C and substance use, when compared to industry studies [3]. The study was stopped early, at which point approximately 7700 participants had been followed for a median of 5 years.

Cardiovascular disease has leapt to the forefront of HIV management, as antiretroviral therapy becomes safer and more potent, and as people living with HIV (PWH) successfully age towards the general population's average life expectancy [4−7]. That said, people with controlled HIV still have an approximately two‐fold overall additional risk of heart, stroke and other major vascular diseases, a by‐product of what is believed to be HIV‐induced inflammation, prior antiretroviral metabolic effects, and associated lifestyle issues like smoking, substance use and mental health problems [8, 9]. Low‐ and‐ middle‐income countries (LMICs) are predicted to bear the brunt of this cardiovascular burden, as diet and lifestyle changes facilitate a rapid transition into an obesity and diabetes epidemic, a concern in HIV high‐burden environments where risk factors for MACE such as hypertension and diabetes are undertreated [10, 11]. HIV remains an exclusion criterion in many cardiovascular and other studies, despite the remarkable benefits in morbidity and mortality gains with modern antiretroviral therapy [12].

## 2 DISCUSSION

So, what did the REPRIEVE study teach us about cardiovascular disase (CVD) risk prediction among PWH? First, major cardiovascular events were significantly lower in the pitavastatin arm, a benefit that increased in parallel with their MACE risk and elegantly presented in a supplement to the final article, as assessed by the American Heart Association and American College of Cardiology 2013 Pooled Cohort Equation risk calculator [2]. In the overall study population, 100 PWH would need to be treated for 5 years to prevent one case of MACE, but the number needed to treat drops to 34 if confined to only higher risk (>10% risk score) PWH and increases to >130 in those with Atherosclerotic Cardiovascular Disease (ASCVD) risk of <5%.

Regarding the safety concerns, those in the pitavastatin group had a higher risk of developing diabetes (mainly in those with pre‐existing risk factors) and a small increase in myalgias and myopathies at a level seen with other statin studies, but low numbers of discontinuations [13, 14]. Over 5 years, there would be one extra case of diabetes for approximately every 75 PWH who were treated.

There were calls for immediate HIV guideline changes to include pitavastatin after the release of the REPRIEVE results [15]. Many guidelines have begun accommodating the REPRIEVE findings.

There are reasons to be cautious in extrapolating the REPRIEVE data too broadly, which was largely conducted in high‐income countries. Less than a third of enrolled participants were women; less than half came from LMICs, with only approximately 15% from Africa, where the HIV epidemic is largely centred, although the local trialists did an admirable job enrolling participants representative of their epidemic. Participants in lower‐income countries were followed for the least amount of time, and understandably had the least number of events. In the sub‐Saharan African cohort recruited into REPRIEVE, there were only eight events in the initial publication of data (five in the placebo arm, three in the pitavastatin arm, from 1157 participants from the region). The authors have published the Pooled Cohort Equation risk calculator overpredicted risk in REPRIEVE in low‐income environments (and underpredicted this in high‐income areas) [16].

Despite these limitations in generalizability, the study deserves close attention. Future gains in metabolic safety and potency of modern antiretroviral regimens will likely be modest because current treatments offer rapid viral suppression and minimal impact on laboratory‐measured cardiovascular risk markers. Moreover, although the impact of modern regimens on treatable clinical factors such as blood pressure and weight gain remains a subject of controversy, the selection of regimens is unlikely to control these alone [17]. The reduced incidence of MACE in REPRIEVE was greater than that expected from reductions in low‐density lipoprotein (LDL) alone, renewing interest in the possible “off‐target” impact of statins on inflammation and plaque stabilization, with continued rich physiology data flowing from the study [18]. That ongoing low‐level inflammation may drive increased cardiovascular risk in PWH, even if stably virally suppressed on antiretroviral therapy, suggests an intriguing interaction between underlying HIV and cardiovascular pathophysiology [19]. As Paul Sax points out in his blog reaction to the study, the transition from infectious diseases doctors to endocrinologists and cardiologists leading breakthroughs in the HIV world demonstrates the remarkable and welcome transition to a focus on life expectancies rapidly approaching normal on current antiretroviral therapies [20].

Several considerations deserve reflection when discussing REPRIEVE results and their implications for guidelines:

### 2.1 Implementation complexity

Additional work is needed to better determine who best benefits from a statin among PWH. HIV care programmes may have problems implementing a screening tool as complex as that used in REPRIEVE. Even a simplified version using only total cholesterol, such as developed by WHO for global use, requires a blood draw or point‐of‐care capacity, and hence a cascade of laboratory resources or equipment support, clinician interpretation and action in prescribing, all interventions that have proved challenging for other tests within HIV programmes, including viral loads and creatinine screening [21]. This speaks to the need for evidence‐based non‐laboratory‐based risk scores, or simple triggers based on easily implemented criteria (age, the presence of risk factors such as hypertension, smoking) that can guide therapy decisions. Qualitative assessments based on these criteria may be sufficient for starting treatment without requiring precise quantitative calculations of MACE risk.

### 2.2 Differing geographic burden of statin‐responsive disease

The geographic sub‐cohort effect sizes suggest that statin use for PWH might not be “one size fits all.” In Southern Africa, for example (supported by the eight cases in the REPRIEVE data, and the fact that the updated report demonstrated little impact on heart failure), ischemic cardiac events appear to be less common, with cardiac failure and pericardial disease being more prevalent [22]. It is unclear whether statins would impact this epidemiology. In addition, diabetes rates are escalating in many African countries, making statin‐induced hyperglycaemia a consideration, even though the risk increase is modest [23, 24]. Thus, the already complicated calculus of benefits versus harms may also be context‐dependent and vary geographically. Would a separate study be needed in this region to convincingly demonstrate benefit? Finally, recent data on the impact of novel agents such as glucagon‐like peptide‐1s and the sodium‐glucose cotransporter‐2 inhibitors, both initially purposed for diabetes but now with impacts on obesity, heart failure, kidney dysfunction and a range of other end‐organ complications, may mean other drug classes are also relevant, despite currently being far more expensive than statins [25].

### 2.3 Screening and treatment for diabetes

Given the predictable increase in the incidence of diabetes in people receiving pitavastatin in the trial, regular glucose screening may be needed to be incorporated with other monitoring. Delayed recognition of diabetes will itself lead to additional cardiovascular events in the long term, although statin use will itself mitigate this risk. This is worrying, as health systems have demonstrated weakness in screening for chronic diseases. Data from separate studies point to gross undertreatment of even simple hypertension within HIV clinical trials in Africa [26]. Even in high‐income countries, chronic diseases in HIV care environments are underdiagnosed and undertreated [27, 28]. It is unclear whether cessation of statin use will reverse glucose intolerance or diabetes (current guidelines, including WHO, suggest continuing statins once diabetes is diagnosed). Lifelong therapies for diabetes remain a challenge in much of the world, especially where access to hypoglycaemic medication is not assured [29].

### 2.4 Cost‐effectiveness

Pitavastatin is available in generic form in some areas of the world, although costs >$300/month in the United States [30]. Plausibly, the MACE prevention benefits extend to other less costly statins, and most clinicians would probably prescribe locally available options, rather than wait for a generic option or comparator study results. The cost‐effectiveness of statin use in this population will need to be calculated across different geographies and scenarios, conscious that different statins and statin intensity have different MACE reduction levels in other studies.

### 2.5 Co‐funding support

Funding for statin and other cardiovascular prevention services for PWH remains a major, unanswered challenge. In most donor‐supported programmes such as PEPFAR and the Global Fund which are the backbone in most low‐income countries, there is typically little to no support for wrap‐around services, such as contraception programmes or chronic disease management such as hypertension or diabetes. In much of Africa, Asia and South America, even in middle‐income countries, these are funded out‐of‐pocket. It seems improbable that support for laboratory screening and statins (or diabetes medications) will be forthcoming when these other impactful interventions are not supported. On the other hand, studies like REPRIEVE may put pressure on these provision systems and may trigger the needed implementation research and resources required for programmes.

### 2.6 Health worker time, resource use and opportunity costs

The effort and focus on averting a relatively small number of cardiovascular events by implementing a novel intervention needs to be carefully weighed against the use of similar resources for other health priorities among PWH, such as smoking reduction, sexual health and contraception, treatment adherence, screening for depression, addressing substance use, taking blood pressure properly and a host of other, currently often poorly implemented interventions. In addition, in overburdened health systems, health workers may interpret a statin implementation programme as another yet thing to add to their workload. If vital measurement taking and monitoring is inadequate for a variety of operational reasons, then one will need to ask who is realistically going to screen and monitor for statin initiation and monitoring and thus national programmes may not buy into it. Again, priority setting (e.g. modelling may assist to set thresholds that trigger assessments) may allow for a more directed use of resources.

### 2.7 Recommendations for future research

Guidelines routinely recommend future research, and REPRIEVE has already published many novel findings, a wealth of information to guide the next wave of studies. Cardiovascular studies are notorious for the length of time they take and their scale, so careful thought will be needed on how the next generation of studies are structured. We recommend:

- Cardiovascular risk prediction scoring updated for LMICs to determine who most benefits from statins, as well as other interventions.
- Relatedly, simplification of candidate selection tools to identify candidates for statin use.
- Well‐designed cost‐effectiveness analysis to determine the individual and health system impact over long time horizons.
- Implementation science programmes to determine the acceptability, feasibility and sustainability of statin integration programmes, with attention to coformulations, monitoring tools and education.

### 2.8 Availability of future alternatives

Although largely untested in HIV populations, and as mentioned above, the pleiotropic effects of the incretin molecules, with impact on mortality and recently a range of other outcomes in those with overweight and heart failure, and similar anti‐inflammatory effects to statins, may make these medications, which do not cause (indeed, they treat) diabetes, more suitable agents, especially if oral formulations can be made to work and side effect profile concerns resolved. However, a trial of the same magnitude and global reach as REPRIEVE is likely needed to persuade decision‐makers. In addition, cost, patents, availability, acceptability, persistence, efficacy within populations with HIV and a host of other important questions require answering before these can be considered.

Guideline committees have already engaged with REPRIEVE data [31]. Implementation will require thoughtfulness by both programmes and clinicians, and active engagement with people with HIV, to have a meaningful discussion about the relatively small benefits, unless applied in high‐risk PWH (who may well be eligible for a statin in many cases), and equally small harms of the intervention. It may be that we require additional data before it is broadly applied across the globe.

## 3 CONCLUSIONS

We will have done nothing if we continue to tie ourselves in debates about whether REPRIEVE's intervention is for everyone. If a REPRIEVE‐2 was released tomorrow with data more generalizable to LMICs, showing significant benefit and addressing all the concerns above, we would be ill‐prepared to implement its findings. Statins are safe, cheap and readily available, as are antihypertensives and diabetes medications. However, statins and antihypertensives are grossly under‐prescribed for primary and secondary prevention, especially in LMICs [26, 32]. The new incretin anti‐obesity medications and a host of similar medications are all highly effective, safe and usable in primary care, with accumulating data on favourable primary and secondary MACE outcomes, and probably affordable once in generic manufacturing hands, albeit awaiting long‐term safety data in some cases. They are set to transform our patient's lives and their cardiovascular future—but something needs to first change in healthcare worker prescribing behaviour.

The starting point perhaps is to begin preparing HIV programmes for something many clinicians have known for a long time: that confining HIV medicine to handing out antiretroviral therapy was always a tiny part of the journey to a long and healthy life. With very safe and potent current therapies, and long‐acting antiretrovirals on the horizon, we need to focus attention on care delivery systems that address both lifestyle and psychosocial support, but also rapid implementation of evidence‐based pharmaceutical interventions that studies like REPRIEVE support [28].

Experience has demonstrated that patients are keen to understand their health, and that HIV clinics can be a place where cardiovascular risk factors can be effectively addressed [33]. Hard questions need to be asked of any HIV clinic or programme, whether it be in a high‐, middle‐ or low‐income country, that is not able to screen and treat common chronic conditions effectively. REPRIEVE and the studies that are sure to come next, raise the bar for care, and PWH will need a health service that can absorb potentially complex prevention interventions at their core.

## COMPETING INTERESTS

SS, SL‐E and WDFV's unit receives funding from the Bill and Melinda Gates Foundation, SA Medical Research Council, National Institutes for Health, Unitaid, Foundation for Innovative New Diagnostics (FIND), Merck and the Children's Investment Fund Foundation (CIFF), and receives drug donations from ViiV Healthcare, Merck, J&J and Gilead Sciences for investigator‐led clinical studies. The unit does investigator‐led studies with Merck, J&J and ViiV providing financial support and is doing commercial drug studies for Merck and Novo. The unit performs evaluations of diagnostic devices for multiple biotech companies. WDFV receives honoraria for educational talks and advisory board membership for Gilead, ViiV, Mylan/Viatris, Merck, Adcock‐Ingram, Aspen, Abbott, Roche, J&J, Sanofi, Boehringer Ingelheim, Thermo‐Fischer and Virology Education. MKA has received honoraria for advisory board participation by Bayer and Eli Lilly, and a grant to Emory University from Merck.

## AUTHORS’ CONTRIBUTIONS

The manuscript flowed from a discussion between SS, WDFV, JMM‐G, SL‐E and MJS, who did the first draft after the first REPRIEVE data were released, with substantial contributions from the other authors and a rewrite once the updated results were made available.

## FUNDING

WDFV, MKA, MJS and SL‐E are partially supported by UG3/UH3HL156388. SS, SL‐E and WDFV are supported by the SAMRC. AM is partially supported by U24HL154426.

## Data Availability

There is no primary data source.
